# Elevated Levels of Galectin-9 but Not Osteopontin in HIV and Tuberculosis Infections Indicate Their Roles in Detecting MTB Infection in HIV Infected Individuals

**DOI:** 10.3389/fmicb.2020.01685

**Published:** 2020-07-17

**Authors:** Ashwini Shete, Shubhangi Bichare, Vishwanath Pujari, Rashmi Virkar, Madhuri Thakar, Manisha Ghate, Sandip Patil, Annapurna Vyakarnam, Raman Gangakhedkar, Gaowa Bai, Toshiro Niki, Toshio Hattori

**Affiliations:** ^1^ICMR-National AIDS Research Institute, Pune, India; ^2^B. J. Medical College and Sassoon General Hospital, Pune, India; ^3^Interactive Research School for Health Affairs (IRSHA), Bharati Vidyapeeth Deemed University, Pune, India; ^4^Indian Institute of Science (IISc), Bengaluru, India; ^5^Indian Council of Medical Research, New Delhi, India; ^6^Department of Health Science and Social Welfare, Kibi International University, Takahashi, Japan; ^7^Department of Immunology, Faculty of Medicine, Kagawa University, Kita-gun, Japan

**Keywords:** galectin-9, osteopontin, HIV, pulmonary tuberculosis, extrapulmonary tuberculosis, latent tuberculosis, HIV/tuberculosis co-infection

## Abstract

Galectin-9 (Gal-9) and osteopontin (OPN) play immunomodulatory roles in tuberculosis and HIV infections. Evaluation of their levels as well as their interplay with different pro-inflammatory cytokines is critical to understand their role in immunopathogenesis of HIV/tuberculosis co-infection considering the complexity of the disease. Plasma levels of these proteins were measured by ELISAs in HIV-negative individuals with pulmonary (*n* = 21), extrapulmonary (*n* = 33), and latent tuberculosis (*n* = 22) and in HIV infected patients with pulmonary (*n* = 14), latent tuberculosis (*n* = 17), and without tuberculosis (*n* = 41). Levels of pro-inflammatory cytokines were estimated by Luminex assay. Receiver operated characteristic curve analysis was performed to evaluate discriminatory roles of these proteins. Spearman’s correlation analysis was performed with the markers of HIV and tuberculosis disease progression to evaluate their immunopathogenic roles. Gal-9 and OPN levels were higher in HIV uninfected patients with active tuberculosis than with latent tuberculosis. Gal-9 but not OPN levels were higher in HIV infected patients with active tuberculosis than with latent tuberculosis. Area under curve for Galectin-9 was >0.9 in HIV/tuberculosis co-infection and extrapulmonary tuberculosis. OPN and IL-6 levels were higher in patients with severe chest X-ray grade indicating its association with severity of the disease and positively correlated with each other. Stronger positive and negative correlations of Gal-9 levels, respectively, with viral loads and CD4 cell counts in HIV infected patients were observed than OPN levels indicating their association with HIV disease progression. Thus, significantly elevated Gal-9 levels were reported for the first time in HIV/tuberculosis co-infection and extrapulmonary tuberculosis in our study than single infections with HIV and tuberculosis. The study indicated a need for further evaluation of monitoring role of Gal-9 for detection of developing tuberculosis in HIV infected individuals. The findings also indicated differential roles of Gal-9 and OPN in the pathogenesis of tuberculosis and HIV infections.

## Introduction

Galectin-9 (Gal-9) and osteopontin (OPN) are matricellular proteins (MCPs) which play an important role in inflammation and tissue remodeling by acting as connective tissue growth factors. MCPs are mainly secreted in extracellular environment during development stages, wound healing, and in response to injury and stress (Bornstein and Sage, 2002; Elola et al., 2007). They also serve as soluble ligands for different cell receptors influencing diverse cellular processes. These proteins play pro-inflammatory as well as immunoregulatory roles in infectious diseases influencing their course. Gal-9 has been shown to play such a dual role in infections depending on the stage of infections. It exerts pro-inflammatory effect through innate immune system during early infection and immune-suppressive effects during chronic stages of infection by inducing apoptosis of activated T cells (Machala et al., 2019b). Gal-9 and Tim3 are shown to act as co-inhibitory receptors for Th1 cells through their interactions leading to apoptosis of these cells (Anderson et al., 2016; Compagno et al., 2020). Although it causes suppression of systemic protective T cell responses, it has been shown to reduce immunopathology at local levels in viral infectious diseases (Sehrawat et al., 2009; Machala et al., 2019b). It has also been shown to restrict entry of several viruses like HIV and CMV in their target cells (Elahi et al., 2012; Machala et al., 2019a). Although interaction of Gal-9 with Tim-3 was shown to induce resistance to HIV-1 infection (Elahi et al., 2012), its interaction with protein disulfide isomerase was shown to enhance HIV-1 entry in CD4 cells (Bi et al., 2011). OPN has been shown to induce tissue damage in many diseases including viral infections (Paul et al., 2017; Sampayo-Escobar et al., 2018) making OPN an attractive target to limit tissue injury (Hirano et al., 2015; Pascapurnama et al., 2017). Conversely, higher OPN expression induced by IRF8 downregulation in tumor cells was found to suppress the activation of CD8 cells by binding to CD44 receptors expressed by them. Hence, it was suggested that OPN may also act as an immune checkpoint (Klement et al., 2018; Shurin, 2018). Therefore, both proteins have a potential to act as co-inhibitory molecules against different target cells.

Plasma levels of these proteins have been shown to be associated with the severity of global diseases, such as dengue, tuberculosis and malaria (Koguchi et al., 2003; Chagan-Yasutan et al., 2013; Dembele et al., 2016). Hence, it is imperative to understand their pathological roles in infectious diseases. In this study, we have measured their plasma levels in HIV and/or tuberculosis (TB) infected individuals and assessed their associations with severity of the diseases. We also looked for their discriminatory activity in a complex disease like HIV/TB co-infection which constitutes one of the biggest public health issues around the world. HIV infected patients have 5–10 times more risk of developing tuberculosis than HIV-uninfected individuals in spite of their treatment with anti-retroviral drugs (Lawn et al., 2006). In 2016, there were an estimated 1 million new TB cases among people who were HIV-positive with 374,000 deaths worldwide (World Health Organization, 2019). Dramatic increase of HIV infection among TB patients was recently reported also in Asia (Solante et al., 2017). Both these infections involve chronic immune activation, ultimately responsible for immunopathogenesis of the diseases. Immune activation has been shown to be the central mechanism responsible for disease progression in HIV infection (Lawn et al., 2001). Immunosuppressive state induced by HIV infection has been shown to hinder immune responses in tuberculosis infection affecting granuloma formation and fibrosis in HIV/tuberculosis co-infected patients. Hence, although these patients are at a higher risk of developing extrapulmonary tuberculosis (EPTB) (Mohammed et al., 2018), the extent of lung damage in pulmonary tuberculosis (PTB) is limited in them (Swaminathan et al., 2007; Stek et al., 2018). Furthermore the co-infected patients are at risk of developing TB-associated immune reconstitution inflammatory syndrome (TB-IRIS) in nearly half of the cases (48%) (Hattori et al., 2016; Lai et al., 2016) highlighting importance of understanding immunopathogenesis of this co-infection.

HIV-infected individuals on ART were shown to exhibit significantly higher Gal-9 expression on both CD4+ and CD8+ T cells compared with ART naïve patients and long term non-progressors (LTNPs) indicating presence of generalized hyperimmune activation in HIV patients despite ART (Shahbaz et al., 2020). It was also reported that the expression of Gal-9 and Tim3 molecules was upregulated during an Mtb infection and that Gal-9/Tim3 pathway was necessary to activate bactericidal mechanisms to control intracellular bacterial growth (Jayaraman et al., 2010). On the contrary, Lipoarabinomannan exposure was found to lead to deceased Gal-9 expression on macrophage favoring Mtb growth (Chavez-Galan et al., 2017). OPN was shown to tip the balance of myeloid and lymphoid populations in mice (Kanayama et al., 2017) and was found to be positively associated with neutrophils numbers and negatively with lymphocyte numbers in TB patients (Shiratori et al., 2016). These findings point to the need to evaluate immunopathogenic roles of OPN and Gal-9 in HIV/TB co-infections.

Both these infections show increased plasma levels of Gal-9 and OPN as reported in different studies (Chagan-Yasutan et al., 2009; Tandon et al., 2014; Hasibuan et al., 2015). However, the data on their levels in HIV/TB co-infections is scarce. They have also been shown to interact with other inflammatory cytokines (Matsuura et al., 2009; Clemente et al., 2016). Hence it is important to study their associations with these cytokines to understand immunopathological mechanisms involved in causing disease progression. It is very important to diagnose the disease status in TB infection for ensuing strategies for timely management and control of the disease. Biomarkers for discriminating active TB from latent TB infection (LTBI) are critical and hence are being focused in many studies. A recently published study evaluated Matrix Metalloproteinases and Tissue Inhibitors of Metalloproteinases for differentiating PTB and EPTB from LTB (Kathamuthu et al., 2020). However, such studies in HIV infected individuals are sparse. Although there are a number of reports showing OPN and Gal-9 elevations in active tuberculosis from different countries, reports regarding their levels in latent infection show discordances (Hasibuan et al., 2015; Shiratori et al., 2016). In this study, we have evaluated the plasma levels of these proteins in LTBI and TB patients to know if this discordance could be resolved or not. Furthermore, both HIV and TB patients show asymptomatic infection for a long time. Development of TB in HIV infected individuals need to be diagnosed at the earliest for its timely management. Since HIV infected patients are likely to present with sputum negative PTB or EPTB (Swaminathan et al., 2010; Rosso et al., 2011), their diagnosis is always challenging. Hence we evaluated these proteins to determine if they can be used as biomarkers indicating development of active tuberculosis in HIV infection.

## Materials and Methods

### Study Population

The study was conducted at ICMR-National AIDS Research Institute (NARI), India after approval of the study protocol by ICMR-National AIDS Research Institute Ethics Committee (Protocol No: NARI-EC/2017-13). Patients were enrolled from NARI and Sassoon General Hospital (SGH). All the procedures were conducted in accordance with the Declaration of Helsinki.

Study participants from different groups and their characteristics were as mentioned in Table 1. The participants included HIV negative individuals with LTBI (HIV^–^LTBI^+^), HIV negative individuals with active pulmonary tuberculosis (HIV^–^PTB^+^), HIV negative individuals with extrapulmonary tuberculosis (HIV^–^EPTB^+^). Antiretroviral therapy (ART) naive HIV-infected individuals with active pulmonary tuberculosis (HIV^+^PTB^+^), with and without latent tuberculosis (HIV^+^LTBI^+^ and HIV^+^LTBI^–^, respectively) were also included. HIV infected patients with CD4 less than 350 cells/mm^3^ and those with HIV/TB co-infection were subsequently initiated on ART as per the national guidelines. LTBI in the study participants was determined by Interferon Gamma Release Assay (IGRA) by a commercially available kit (T-SPOT.TB, Oxford Immunotec Ltd, United Kingdom) as described previously (Shete et al., 2018). HIV^–^PTB patients were divided radiologically into two categories; mild to moderate and severe. Lesions involving four or more zones without cavities or more than three zones with cavities were categorized as the severe tuberculosis and the rest were categorized as non-severe tuberculosis (Dholakia et al., 2012). Blood samples of these patients were collected before putting them on anti-tuberculous therapy and were used for PBMCs and plasma separation. CD4 cells were quantified by Flow cytometry by using TruCount Kit (Becton-Dickinson, United States) and HIV viral loads (Abbott laboratories, United States) were estimated on the samples of HIV infected patients as per manufacturer’s instructions.

**TABLE 1:** Information of study groups and participants.

Groups	HIV- LTBI+ (*n* = 22)	HIV-PTB+ (*n* = 21)		HIV-EPTB+ (*n* = 33)	HIV+PTB+ (*n* = 14)	HIV+LTBI + (*n* = 17)	HIV+ LTBI- (*n* = 41)
		Non-severe (*n* = 10)	Severe (*n* = 11)				
Age (years): Median (range)	35 (19–45)	27.5 (17–65)	28 (18–63)	30 (16–60)	39 (22–60)	36 (24–56)	37 (27–53)
Sex (M:F)	16:6	9:	7:4	22:11	10: 4	5:12	15:26
CD4 (cells/mm^3^): Median (range)	Not done	Not done	Not done	Not done	150 (80–525)	308 (148–1565)	335 (19–1207)
CD4%:Median (range)	Not done	Not done	Not done	Not done	12 (8–22)	17 (7–46)	22 (2–49)
Viral load (copies/ml): Median (range)	Not done	Not done	Not done	Not done	251540 (1155–434510)	458227 (46816–1606720)	14340 (115–4963079)
IGRA positivity	22 (100%)	Not done	Not done	Not done	Not done	17 (100%)	0 (0%)
Organs involved	Not applicable	Lungs	Lungs	Pleural effusion (23), abdominal (4), spine (1), miliary (2), cold abscess (3)	Lungs	Not applicable	Not applicable

### ELISA

Plasma concentrations of OPN and Gal-9 were measured using the Human Osteopontin and Galectin-9 DuoSet ELISA Kits (R&D Systems, United States). The kit has been shown to detect full length as well as degraded products of Gal-9 [29]. The ELISAs were performed according to manufacturers’ manuals after diluting plasma samples to 1:100 and 1:10 by reagent diluent supplied with the kits for OPN and Gal-9 ELISAs, respectively. Concentrations of the MCPs in the samples were determined by plotting standard curve as per the manufacturer’s instructions. Limit of detection (LOD) for the OPN and Gal-9 ELISAs were 62.5 and 93.8 pg/ml, respectively.

### Luminex Assay

Pro-inflammatory cytokines levels were estimated in plasma samples of the participants by Luminex assay (Bio-Rad, United States) as per the manufacturer’s instructions. The cytokines evaluated were IL-6, IL-8, IL-10, IFN-γ, IP-10, and TNF-α. The concentrations of the cytokines were calculated as per manufacturer’s instructions.

### Data Analysis

GraphPad Prism software was used to perform statistical analysis and plotting graphs. Multiple comparisons were assessed through Kruskal–Wallis test with Dunn’s multiple comparison testing. Spearman’s nonparametric test was performed for correlation analyses. *P*-values less than 0.05 were considered significant. easyROC: a web-tool (ver. 1.3.1) was used for receiver operating characteristic (ROC) analysis.

## Results

### Gal-9 Levels in Different Groups of Study Participants

**FIGURE 1:**
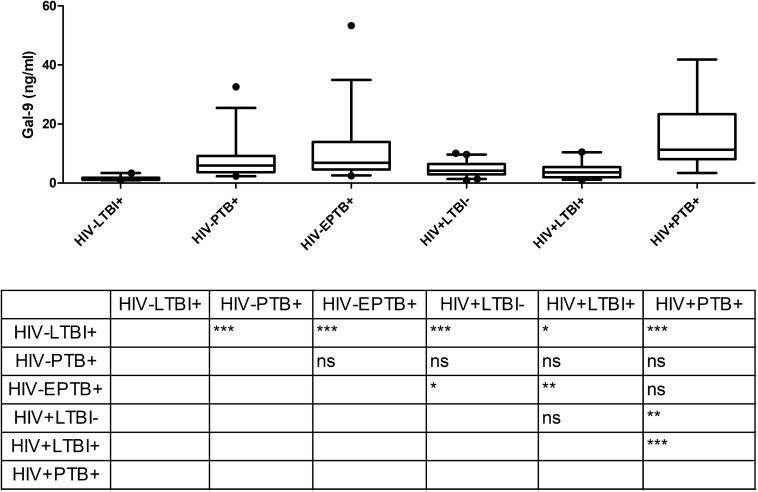
Gal-9 levels in plasma samples of study participants: The figure shows plasma levels of Gal-9 (ng/ml) plotted on *Y* axis from participants of different study groups (*X* axis). Medians values and interquartile ranges for the groups are plotted as bars and error bars. Multiple comparisons were assessed through Kruskal–Wallis test with Dunn’s multiple comparison testing. Levels of significance between different study groups are shown in the Table embedded in the figure. *P* values ≤ 0.05, ≤ 0.01 and ≤ 0.001 are indicated as *, ** and ***, respectively.

To understand differences in elevations in various clinical situations Gal-9 levels in patients’ plasma were measured by ELISA and are shown in Figure 1. The levels were significantly higher (*P* < 0.0001) in HIV^–^PTB^+^ group (Median: 5.9 ng/ml; range: 2.3–32.5 ng/ml) as compared to HIV^–^LTBI^+^ group (Median: 1.19 ng/ml; range: 0.93–3.37 ng/ml). HIV^+^PTB^+^ co-infected patients (Median: 11.29 ng/ml; range: 3.36–41.8 ng/ml) had significantly higher levels of Gal-9 than HIV^–^LTBI^+^, HIV^+^LTBI^–^ (Median: 4.1 ng/ml; range: 0.95–10.06 ng/ml) and HIV^+^LTBI^+^ groups (Median: 3.5 ng/ml; range: 1.12–10.46 ng/ml). HIV^–^LTBI^+^ patients had significantly lower levels of Gal-9 than HIV^+^LTBI^+^ group and HIV^+^LTBI^–^ groups. HIV^–^EPTB^+^ (Median: 6.8 ng/ml; range: 2.4–53.3 ng/ml) also had significantly higher levels of Gal-9 as compared to HIV^–^LTBI^+^ group. But there was no significant difference in HIV^–^PTB^+^ and EPTB^+^ patients.

### OPN Levels in Different Groups of Study Participants

**FIGURE 2:**
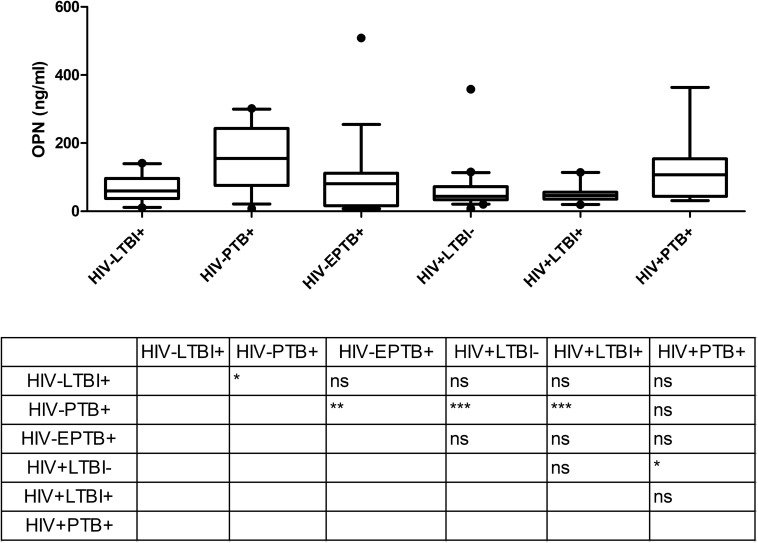
OPN levels in plasma samples of study participants: The figure shows plasma levels of OPN (ng/ml) plotted on *Y* axis from participants of different study groups (*X* axis). Medians values and interquartile ranges for the groups are plotted as bars and error bars. Multiple comparisons were assessed through Kruskal–Wallis test with Dunn’s multiple comparison testing. The table shows levels of significance between different study groups. *P* values ≤ 0.05, ≤ 0.01 and ≤ 0.001 are indicated as *, ** and ***, respectively.

Osteopontin levels in plasma determined by ELISA were significantly higher in HIV^–^PTB^+^ group (Median: 154.7 ng/ml; range: 7.9–301.9 ng/ml) as compared to HIV^–^LTBI^+^ group (Median: 59.2 ng/ml; range: 10.8–141.1 ng/ml) (Figure 2). Contrary to Gal-9, HIV^+^PTB^+^ co-infected patients (Median: 107.0 ng/ml; range: 31.1–363.6 ng/ml) did not have higher levels of OPN than patients with only tuberculosis (HIV^–^PTB^+^). Furthermore, HIV^+^PTB^+^ co-infected patients showed significantly higher levels than HIV^+^LTBI^–^ group (Median: 43.7 ng/ml; range: 7.9–358.2 ng/ml), but not than HIV^+^LTBI^+^ group (Median: 45.5 ng/ml; range: 19.1–114.1 ng/ml; *P* = 0.012). HIV^–^EPTB^+^ patients (Median: 80.5 ng/ml; range: 4.4–509.0 ng/ml) did not have higher levels of OPN as compared to LTBI patients. However, they had significantly lower OPN levels than HIV^–^PTB^+^ patients.

### ROC Curves of Gal-9 and OPN for Diagnosis of Active Tuberculosis in HIV-Infected and Uninfected Patients

**FIGURE 3:**
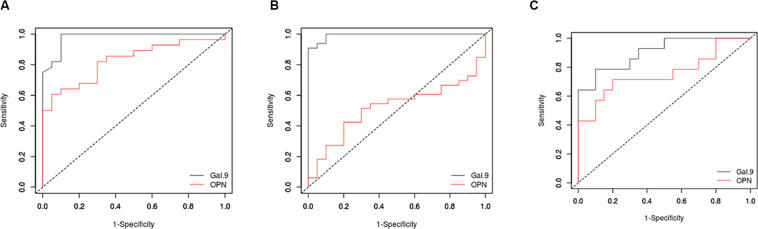
Receiver operating characteristic (ROC) curves for Gal-9 and OPN levels. ROC curves are plotted for Gal-9 (black) and OPN (red) levels in HIV-PTB+ **(A)**, HIV-EPTB+ **(B)**, and HIV+PTB+ **(C)** patients. Sensitivity and 1-specificity are plotted on *Y* and *X* axes, respectively.

To understand discriminatory potential of Gal-9 and OPN levels in active versus latent tuberculosis, ROC analysis was conducted with Gal-9 and OPN data entered against the disease status (1 = patients having active PTB or EPTB, 0 = patients with latent tuberculosis). The curves were drawn by plotting sensitivity on Y-axis and the 1-specificity on X-axis (Figure 3). Area under curve (AUC) values, which represent discriminatory power of Gal-9 in HIV^–^PTB^+^, HIV^–^EPTB^+^, and HIV^+^PTB^+^ were 0.98, 0.99, and 0.90, and those of OPN levels in the same group were 0.83, 0.52, and 0.76, respectively. Youden index, cutoff values of Gal-9 and OPN levels for differentiating between active and latent tuberculosis in these conditions are mentioned in Table 2. AUC, Youden index, sensitivity and specificity for Gal-9 levels were higher than those for OPN levels indicating Gal-9 as a better marker for differentiating between active and latent tuberculosis TB in these patients although higher specificity of OPN in HIV^–^PTB+ was seen. Cut-offs for Gal-9 levels differed among HIV-PTB, HIV-EPTB and HIV+PTB+ patients and that of HIV+PTB showed the highest level (Table 2).

**TABLE 2:** Receiver operating characteristic (ROC) curve analysis.

Group-wise parameters	HIV-PTB+		HIV-EPTB+		HIV+PTB+	
	Gal-9	OPN	Gal-9	OPN	Gal-9	OPN
AUC	0.98	0.83	0.99	0.52	0.90	0.76
Standard Error of AUC	0.02	0.06	0.01	0.08	0.05	0.09
Cut off	2.32	125.38	3.39	97.40	9.14	66.87
Youden Index	0.90	0.55	0.90	0.22	0.60	0.50
Sensitivity	1.00	0.61	0.90	0.40	0.78	0.71
Specificity	0.90	0.95	1.00	0.80	0.90	0.80

### Association of OPN and Gal-9 Levels With the Markers of Disease Severity

**FIGURE 4:**
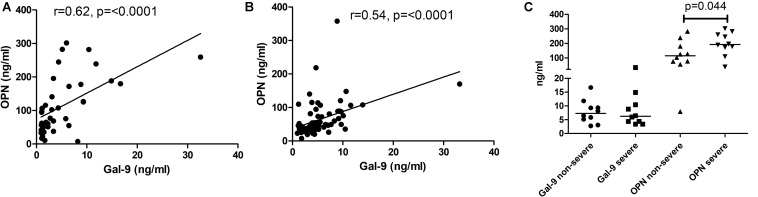
Correlations of matricellular protein levels with the disease severity. Panels **(A,B)** show correlation of Gal-9 levels with OPN levels in HIV uninfected and HIV-infected study participants, respectively. The correlation coefficient (*r*) and *p*-values are also shown in the figure. Panel **(C)** shows Gal-9 and OPN levels in non-severe and severe grade tuberculosis. *p*-values calculated by Mann Whitney test showing significant difference between the groups as are shown in the figure.

To understand the immunopathological roles of Gal-9 and OPN, we have examined the levels for their association with tuberculosis disease severity. OPN levels correlated significantly with Gal-9 levels in the HIV-uninfected (*r* = 0.62, *P* < 0.0001) and HIV-infected (*r* = 0.54, *P* < 0.0001) study participants (Figures 4A,B). Levels of OPN, but not Gal-9, were significantly higher (*P* = 0.04) in patients with severe grade chest lesions as compared to those with non-severe grade lesions (Figure 4C).

### Association of Pro-inflammatory Cytokine Levels With Tuberculosis Disease Severity and With OPN and Gal-9 Levels in HIV-Uninfected Pulmonary Tuberculosis Patients

We also analyzed levels of different pro-inflammatory cytokines for their association with tuberculosis disease severity (Table 3). LOD for the cytokines are also mentioned in the Table 3. Although levels of all estimated pro-inflammatory cytokines were significantly higher in plasma samples of HIV^–^PTB^+^ patients in comparison to HIV^–^LTBI^+^ group, only IL-6 levels were significantly higher (*P* = 0.04) in patients with severe grade chest lesions as compared to those with non-severe grade lesions. Positive correlations were seen between IL-8, IP-10 and Gal-9; IL-6, IL-8 and OPN levels in PTB patients.

**TABLE 3:** Levels of pro-inflammatory cytokines and their correlations with tuberculosis disease severity.

Cytokines (LOD)	HIV^–^LTBI^+^ Median (ranges): pg/ml	HIV^–^PTB^+^ Median (ranges): pg/ml	*P*-value of HIV^–^LTBI versus HIV^–^PTB	*P*-value of non-severe versus severe HIV^–^PTB	Correlation with Gal-9 *r*(*p*)	Correlation with OPN *r*(*p*)
IL-6 (0.34 pg/ml)	20.97 (3.54–46.48)	156.6 (23.5–864.9)	<0.0001	0.044	0.3774 (0.0504)	0.3985 (0.0409)
IL-8 (0.36 pg/ml)	56.34 (50–190.25)	283.7 (50–431.9)	0.0001	0.25	0.4289 (0.0296)	0.40 (0.03)
IL-10 (0.69 pg/ml)	6.01 (3.57–8.51)	11.96 (6.43–992.3)	<0.0001	0.2	0.2426 (0.1514)	0.1522 (0.2609)
IFN-γ (1.05 pg/ml)	800 (800–1463.8)	4214 (800–5458)	<0.0001	0.96	0.2549 (0.1391)	0.1435 (0.2731)
IP-10 (1.43 pg/ml)	3562.59 (400–9862.29)	19273 (5598–42326)	<0.0001	0.83	0.4211 (0.0322)	0.3308 (0.0771)
TNF-α (1.13 pg/ml)	Below detection limit	20 (20–534.9)	Not obtained	0.27	0.1844 (0.2183)	0.186 (0.2162)

*LOD, limit of detection.*

### Correlation of OPN and Gal-9 Levels With the Markers of HIV Disease Progression

**FIGURE 5:**
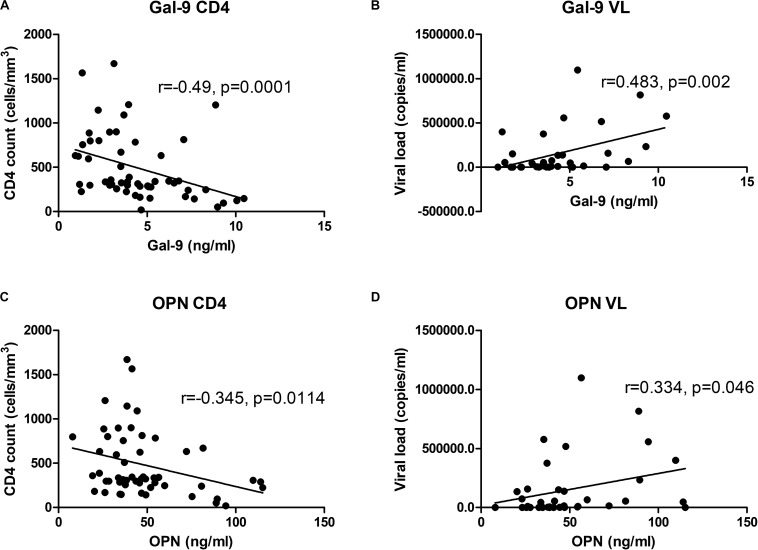
Correlations of Gal-9 levels and OPN levels with CD4 cell counts and viral load: Correlations of Gal-9 and OPN levels with CD4 cell counts in HIV infected patients are shown in **(A,C)** parts of the figure, respectively. Whereas correlations of Gal-9 and OPN levels with viral loads in HIV infected patients are shown in the parts **(B,D)**, respectively. Gal-9 levels and OPN levels are plotted on *X* axis. CD4 counts/viral loads are plotted on *Y* axis. The correlation coefficient (*r*) and *p* value as assessed by Spearman test are mentioned in the figure.

To understand why Gal-9 levels showed higher levels and lower AUC in HIV^+^PTB^+^ than HIV-PTB^+^, and higher AUC than OPN, we asked if Gal-9 levels are also influenced by markers representative of HIV disease progression like CD4 counts and HIV viral loads. We found that the levels correlated more strongly with the markers of HIV disease progression than OPN levels. Gal-9 and OPN levels showed significant negative correlation with CD4 counts (*r* = −0.49 and *r* = −0.345, respectively). They also showed significant positive correlation with viral loads (*r* = 0.483 and *r* = 0.334, respectively) of the HIV infected patients without active tuberculosis (Figure 5). Both of these findings support that Gal-9 levels showed stronger correlation with representative HIV progression markers than OPN levels.

## Discussion

Gal-9 and OPN having immunomodulatory functions act as immune check inhibitors and play an important role in many infectious as well as non-infectious diseases. In this study, plasma levels of Gal-9 and OPN were estimated in HIV and tuberculosis infections to determine if they can be used as markers for developing tuberculosis in HIV-infected individuals. We also assessed if their levels reflect disease severity or not. Gal-9 levels were higher in PTB as well as EPTB as compared to latent tuberculosis. High levels of Gal-9 were reported in pleural fluid in a patient with tuberculous pleural effusion (Zhao et al., 2017), indicating possible production of Gal-9 at the site of inflammation. Higher AUC values of Gal-9 levels in both PTB and EPTB indicated its value in the detection of development of active tuberculosis from latent tuberculosis irrespective of the organ involvement. However, the findings need to be confirmed with larger sample size. Gal-9 levels in our study showed higher AUC values than the previously reported values using full length Gal-9 protein detection (Shiratori et al., 2016). We also observed significantly elevated levels of Gal-9 in PTB individuals than LTBI in contrast to an earlier report where no such difference was observed, which involved patients within the first 2 weeks after therapy (Hasibuan et al., 2015). Reduction in Gal-9 levels after initiation of antitubercular treatment needs to be investigated further to determine the monitoring potential of Gal-9 levels in HIV/TB co-infection as was demonstrated in acute infections such as dengue and malaria (Chagan-Yasutan et al., 2013; Dembele et al., 2016).

While there are reports on Gal-9 suppressing HIV infectivity, other reports demonstrated Gal-9 (Elahi et al., 2012) to enhance HIV transcription through T-cell receptor (TCR)-based ERK signaling as well as to increase its infectivity through indirect pathway (Bi et al., 2011; Colomb et al., 2019). Elevated Gal-9 levels in chronic HIV infection have been shown to correlate with markers of HIV disease progression (Jost et al., 2013; Tandon et al., 2014). We also found increased Gal-9 levels even in single HIV infection and the levels correlated positively with viral load and negatively with CD4 count indicating its association with HIV disease progression. Further, Gal-9 levels were higher in HIV^+^PTB^+^ co-infected patients than single HIV (HIV^+^TB^–^ group) and tuberculosis (HIV^–^PTB group) infections indicating synergistic effect of both infections in increasing Gal-9 levels. Although it is difficult to delineate a single mechanism for enhanced Gal-9 synthesis in HIV/TB patients, it is apparent that TCR-dependent ERK signaling after Gal-9 stimulation may be involved in HIV replication in HIV/TB infection. Brefelamide and its derivatives, which are shown to inhibit Gal-9 through ERK inhibition, might help to halt the vicious cycle caused by these proteins and ameliorate symptoms of this deadly co-infection (Kubohara and Kikuchi, 2018; Bai et al., 2019).

Contrary to Gal-9, OPN levels in our study were elevated in PTB as compared to LTBI, but not in EPTB and HIV^+^PTB^+^. Higher OPN levels in PTB patients as compared to LTBI individuals had been reported earlier (Hasibuan et al., 2015). OPN levels were not higher in HIV^+^PTB^+^ group as compared to HIV^+^LTBI^+^ group. OPN has been shown to be induced by IFN-γ (Li et al., 2003) and associated with Th1 responses (Shinohara et al., 2005). Since HIV infection has been associated with immunosuppressive condition, levels of OPN might not have increased further in these patients. In contrast, OPN levels had been shown to be higher in extensively HIV/tuberculosis co-infected patients than those with only active PTB (Ridruechai et al., 2011). This may be due to extensive nature of the disease reported in these patients. OPN is also reported to be involved in granuloma formation in HIV^–^PTB patients, which is usually less predominant in HIV^+^PTB patients (Shiratori et al., 2016). These findings indicate that OPN may not be useful to detect TB development in HIV^+^ patients.

Severity of PTB has been shown to be mediated by immune mechanisms (Kumar, 2016) and it has been shown that HIV infected patients having lower CD4 counts usually present with non-cavitatory tuberculosis (Padyana et al., 2012). We assessed association of Gal-9 and OPN levels with disease severity in immune-competent HIV-negative individuals. OPN along with IL-6 were found to be associated with disease severity in PTB patients indicating their role in immunopathogenesis of PTB. OPN levels have been shown to be associated with tuberculosis disease severity in earlier studies (Koguchi et al., 2003; Padyana et al., 2012). IL-6 has also been shown to mediate immunopathogenesis in tuberculosis infection and contribute to the disease severity (Jost et al., 2013; Merani et al., 2015). IL-6 and OPN have been shown to upregulate each other and can have additive pro-inflammatory effect (Li et al., 2003; Shinohara et al., 2005). OPN levels were not raised in EPTB in our study, which has been thought to represent a milder clinical form of the disease (Swaminathan et al., 2007; Kumar, 2016; Stek et al., 2018).

Correlation between Gal-9 and OPN levels was reported previously in HIV^–^PTB patients (Shiratori et al., 2016). We also observed their correlation in HIV infected patients in addition to the uninfected individuals. IL-8 levels were found to correlate positively with Gal-9 and OPN in tuberculosis in our study. Both Gal-9 and OPN have been reported to induce IL-8 (Yang et al., 2014; Akashi et al., 2017) which acts as a chemotactic factor for neutrophils (Henkels et al., 2011) possibly explaining increased influx of neutrophils observed in tuberculosis. We also observed positive correlation between Gal-9 and IP-10 levels. Association of Gal-9 levels with other macrophage derived inflammatory molecules such as IL-8 and IP-10 has been reported previously in infectious diseases indicating its association with macrophage activation (Chagan-Yasutan et al., 2013).

## Conclusion

Finally, our data indicated that Gal-9 levels reflect not only the disease severity of HIV but also could detect development of TB in HIV infection for the first time. Application of Gal-9 as a monitoring tool for HIV/TB co-infection and EPTB needs to be carefully examined with a study on larger numbers of patients. Association of Gal-9 levels with CD4 cell counts and HIV viral load indicated its immunopathogenic role in HIV diseases progression. OPN levels, found to be associated with severity of lung damage, were not elevated in HIV infection suggesting possible mechanism for less severe lung damage or smaller granuloma observed in such patients. The differential immunopathogenic roles of Gal-9 and OPN in HIV and tuberculosis disease progression need to be further investigated for developing monitoring as well as therapeutic strategies.

## Data Availability Statement

All datasets generated for this study are included in the article/supplementary material.

## Ethics Statement

The studies involving human participants were reviewed and approved by the ICMR-National AIDS Research Institute Ethics Committee, Pune. The patients/participants provided their written informed consent to participate in this study.

## Author Contributions

AS and TH conceptualized the study. VP undertook the data curation. AS, SB, RV, AV, GB, and TN undertook the formal analysis. TH was responsible for the funding acquisition. SP contributed to the investigation. SB, VP, RV, and SP contributed to the methodology. AS, VP, MT, MG, AV, RG, and TH undertook the project administration. MT, MG, SP, AV, RG, and TN were responsible for the supervision. AS contributed to the writing (original draft). SB, VP, RV, MT, MG, SP, AV, RG, TN, and TH contributed to the writing (review and editing). All authors contributed to the article and approved the submitted version.

## Conflict of Interest

The authors declare that the research was conducted in the absence of any commercial or financial relationships that could be construed as a potential conflict of interest.
